# A Dentifrice

**Published:** 1899-10

**Authors:** W. J. Wallace

**Affiliations:** Saugerties, N. Y.


ARTICLE III.

### A DENTIFRICE.

DR. W. J. WALLACE, SAUGERTIES, N. Y.

What is offered may not be new, for a wise man of old has told us "there is nothing new under the sun," but it is original in one sense, as I have never seen the same formula in print.

While attending the New York Dental School in 1893-'4, one of the lecturers mentioned the requirements of a dentifrice, but left each student to study out what was necessary, and I judge others are no better instructed.

Dentifrices supplied by the drug stores are generally chalk and some scent, or chalk and carbolic acid, if in powder form. If a paste, castle soop is added. The fluid forms have a decoction of soap bark with aromatics. These are good as cleansing agents, but other things should be considered as well. The formula which I offer is:

| | |
|---|---|
| Precipitated chalk, - - | lb. j |
| Bicarbonate of soda, - - | ℥ iij |
| Cocoa butter, - - - | ℥ ij |
| Oil of cassia - - - | ʒ iv |
| Krameria tincture, - - | ʒ iij. |

The proper mixing of the ingredients is a matter of some importance. Melt the coca butter over steam, as it scorches readily, then add the oil of cassia and krameria, stir well and then incorporate the soda, and lastly, the chalk. After stirring well together, turn out on a piece of heavy sized paper and spatulate thoroughly to work out little lumps that will form. This gives a smooth fine powder. The krameria is simply a coloring, giving a delicate pink. Harmless.

Oil of cassia is a germicide. The cocoa butter being of an oily nature, holds the cassia longer and seems the most pleasant and is also nourishing and soothing to the gums. The soda bicarbonate neutralizes any acid formed by fermentation, and assists to prevent salivary calculus.

This makes a very agreeable dentifrice for regular use, and I have found it especially beneficial in those cases where the gums are congested at the margins, and the teeth commencing to be loosened, hardly to be classed as pyorrheal, more of the nature of subacute or chronic gingivitis. Two or three applications of calendula tincture, and the use of the powder each evening the last thing before retiring, being particular to leave what tooth powder remains about the teeth, has, after a time, restored the gums to a healthy condition and the teeth have become normally firm again.

In the June or July number of "Items of Interest," 1894, I think there is an article from the pen of an English dentist naming most of these ingredients as an "ideal tooth powder"; no proportions or quantities are there given, however.—*Items of Interest.*

